# Hyperglycemia-induced isolated aphasic status epilepticus a case-report and literature review

**DOI:** 10.1590/1980-5764-DN-2024-0177

**Published:** 2024-12-16

**Authors:** Jedsada Khieukhajee, Suphasit Seoratanaphunt, Krittima Asavaveeradech, Sunisa Hangsapruek

**Affiliations:** 1 Neurological Institute of Thailand, Department of Neurology, Memory Clinic, Ratchathewi, Bangkok, Thailand.; 2 Neurological Institute of Thailand, Department of Neurology, Epilepsy Unit, Ratchathewi, Bangkok, Thailand.; 3 Neurological Institute of Thailand, Department of Neuroradiology, Ratchathewi, Bangkok, Thailand.

**Keywords:** Hyperglycemia, Aphasia, Status Epilepticus, Hiperglicemia, Afasia, Estado Epiléptico

## Abstract

Neurological manifestations of nonketotic hyperglycemia are frequently seen, with mainly symptoms of confusion or coma. While hyperglycemia-induced seizures are less common, isolated aphasic status epilepticus is very rare, difficult to diagnose, and may be unrecognized by clinicians. In this case report, a 51-year-old man who presented with confusion and incoherent speech for two weeks is discussed. He was diagnosed with nonketotic hyperglycemia, whose electroencephalography (EEG) findings were suggestive of isolated aphasic status epilepticus. His magnetic resonance imaging (MRI) of the brain showed subcortical T2/FLAIR hypointense lesions with cortical T2/FLAIR hyperintensities and restricted diffusion. Although this condition usually responds well to intensive insulin therapy and fluid replacement, many researchers found some persistent aphasic seizures that did not improve until the addition of anti-seizure medications (ASMs).

## RESUMO

Manifestações neurológicas de hiperglicemia não-cetótica são frequentemente vistas e abrangem muitos sintomas de confusão ou coma. Embora convulsões induzidas por hiperglicemia sejam menos comuns, o estado de mal epiléptico afásico isolado é muito raro, difícil de diagnosticar e pode não ser reconhecido por médicos. Um homem de 51 anos que apresentou confusão e fala incoerente por duas semanas é discutido neste relato de caso. Ele foi diagnosticado com hiperglicemia não-cetótica e os seus resultados de eletroencefalografia (EEG) foram sugestivos do estado de mal epiléptico afásico isolado. A ressonância magnética (RM) de seu cérebro mostrou lesões hipointensas subcorticais T2/FLAIR com hiperintensidades corticais em T2/FLAIR e difusão restrita. Embora essa condição geralmente responda bem à terapia intensiva com insulina e reposição de fluidos, diversos pesquisadores relatam convulsões afásicas persistentes que não melhoraram até a adição de medicamentos anticonvulsivantes (ASMs).

## INTRODUCTION

Acute aphasia is a very common condition that is associated with many neurological diseases such as hypoglycemia, drug abuse, and electrolyte abnormalities. However, hyperglycemia is less frequently associated with aphasia. While repetitive focal motor seizures and epilepsia partialis continua are the common presenting symptoms of nonketotic hyperglycemia^1^, isolated aphasic seizures are less frequent and very difficult to diagnose without advanced investigations^2^. We report a case of hyperglycemia-induced aphasia from non-motor seizures in the dominant cerebral hemisphere. We also review the clinical manifestations, investigations, and management of this condition from previous literature.

## CASE PRESENTATION

A 51-year-old man, right-handed, presented to the outpatient clinic with confusion and incoherent speech for two weeks. He did not have any history of underlying disease or drug abuse. His symptoms started one month prior when his coworkers noticed that the patient was more agitated than usual. He was also more forgetful about job tasks. Two weeks later, he began to have incoherent speech and had difficulty following job orders. He also had word-finding problems that improved when given hints. Two days prior to the visit, he started having visual hallucinations of his neighbors around his house. He did not have any abnormal motor or movement symptoms.

The initial examination showed a patient with a body mass index of 17.36 kg/m^2^. All of his vital signs were normal. The initial neurological examination demonstrated a mildly confused patient. Even though he was able to follow only easy one-step verbal commands, he could simply follow body language commands without any effort. He could also remember mealtimes and attending staff correctly. Thus, delirium was less likely the main issue of his overall symptoms. The language fluency assessment showed an obviously decreased speech rate with frequent utterances and neologisms. He was unable to perform any naming, repetition, reading, and writing tests. The cranial nerve and motor examinations were all unremarkable. His initial plasma glucose was 557 mg/dL. Other laboratory data included serum osmolarity 294 mOsm/L, sodium 129.4 mmol/L, and bicarbonate 27.1 mmol/L. Other electrolyte levels, serum ketone, and arterial blood gas were within normal limits. His hemoglobin A1C was 16.5%. The results of cerebrospinal fluid (CSF) and serum polymerase chain reaction (PCR) panel for viral encephalitis and autoimmune encephalitis were all negative. Therefore, an initial diagnosis of global aphasia and type 2 diabetes mellitus (DM) was suspected.

**Figure 1:**
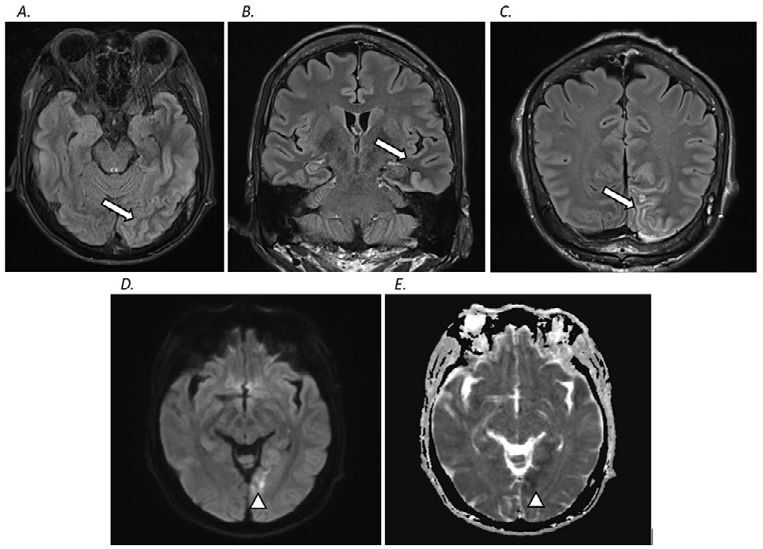
The 3-tesla brain MRI of the patient showed mild cortical hyperintense and subcortical hypointense T2/FLAIR lesions expanding from the left mesial temporal, inferior temporal, and occipital areas (arrows) [FLAIR; A – axial view, B and C – coronal view]. Restricted diffusion was presented at the left mesial temporal area with involvement of the left hippocampal gyrus (arrowheads). [D – DWI axial view, E – ADC axial view].

Abbreviations: FLAIR, fluid attenuated inversion recovery; DWI, diffusion-weighted imaging; ADC, apparent diffusion coefficient.

His magnetic resonance imaging (MRI) of the brain showed cortical hyperintense and subcortical hypointense T2/FLAIR lesions at left medial occipitotemporal gyrus, left occipital, and left medial temporal lobe structures. Minimal restricted diffusion with reduced apparent diffusion coefficient (ADC) was seen at left medial temporal through left medial occipital cortices. No Gadolinium enhancement was seen on the lesion (Figure 1). The presence of extensive vasogenic edematous lesion with minimal restricted diffusion made the possibility of arterial ischemic lesion less likely. No significant lesions in the internal carotid and vertebrobasilar systems were found in the magnetic resonance angiography (MRA) of the brain.

**Figure 2:**
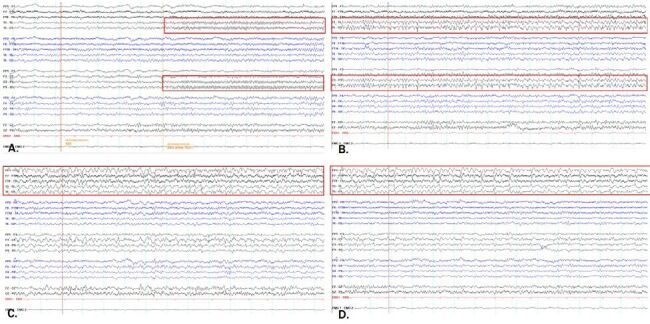
The EEG was recorded with bipolar montage and the filter set to 1-70 Hz using a notch filter of 50 Hz. The sensitivity is 7 μV/mm. The EEG seizure began with low amplitude rhythmic fast activities in the left posterior head region (A). Then, these activities evolved to moderate amplitude theta and delta activities (B) and moderate amplitude sharply contour rhythmic theta activity in the same region (C – D), suggesting EEG seizure arising from the left posterior temporal region (red box). The EEG seizure lasted approximately 120-180 seconds.

Considering the correlation between the abnormal lesions from the brain MRI and his aphasic symptoms, electroencephalography (EEG) was performed to evaluate seizures. The initial EEG showed three electrographic seizures arising from the left posterior temporal area with spatiotemporal evolution. The EEG seizure lasted approximately 120-180 seconds without clinical motor symptoms (Figure 2).

The provisional diagnosis of isolated aphasic status epilepticus from nonketotic hyperglycemia was suggested. Saline infusion and subcutaneous insulin treatment were performed. However, subsequent EEG still showed two subtle electrographic seizures from the same area. Then, intravenous levetiracetam 2,000 mg/day was prescribed. After intensive blood glucose treatment, the patient’s symptoms gradually improved with fluent short-sentence speech and better comprehension. After treatment, his EEG had improved to intermittent theta slowing at the left posterior temporal area without an electrographic seizure.

He was discharged with recovery after 18 days of hospitalization. He came back for follow-up visits at the outpatient clinic for six months without any recurrent symptoms. His recent language examination only found residual mildly impaired long sentence repetition, but normal spontaneous speech, naming, and comprehension. His blood glucose level was controlled within the range of 90-150 mg/dL. Levetiracetam 1,000 mg/day was given for maintenance therapy of seizures due to the patient’s own preference. Finally, it was discontinued one year after disease onset without any recurrent seizures.

### Ethical approval statement

This study was approved by the Institutional Review Board of the Neurological Institute of Thailand (Research No. 66042).

## DISCUSSION

Neurological manifestations from nonketotic hyperglycemia are common, with mainly symptoms of global cerebral dysfunction such as confusion, stupor, or coma. However, focal neurological deficits are occasionally seen with striatal involvement presenting as hemichorea and focal cortical involvement presenting as hemianopia, visual hallucination, seizure, and other stroke-like features^3^. Although hyperglycemia-induced seizures usually manifest as repetitive focal motor seizures and sometimes as epilepsia partialis continua, mainly involving unilateral face and/or limb, other seizure types could also occur^1,4^. Our patient fulfilled the diagnostic criteria of hyperglycemia-induced seizures proposed by Tiamkao et al.^4^ with hyperosmolar hyperglycemic state and seizures that resolved after normalizing plasma glucose and osmolarity.

While convulsive status epilepticus from metabolic disturbances is frequently seen, isolated aphasic status epilepticus without motor seizure is rare and very difficult to diagnose^2^. On literature reviews, reports of isolated aphasic status epilepticus from hyperglycemia are scarce and occasionally do not have clearly documented abnormal EEG and MRI findings (Supplementary Material Table S1 — available at https://www.demneuropsy.com.br/wp-content/uploads/2024/10/DN-2024.0177-Supplementary-Material.docx). However, the clinical course of aphasic features with preserved consciousness and EEG findings of our patient were consistent with the aphasic status epilepticus criteria as defined by Rosenbaum et al.^5^ and modified by Grimes and Guberman^6^.

Although epileptic seizures occur in about 25% of nonketotic hyperglycemia cases, the incidence of isolated aphasic seizures is unknown^7,8^. The most common initial presentation was global aphasia with fluctuating course (with only pure alexia in one report^9^). While some patients may have motor symptoms accompanied by visual disturbances that would point out seizure as a cause, many patients have only isolated ictal aphasia along the entire course of the disease^10^. The most common MRI abnormalities of nonketotic hyperglycemia are subcortical T2/FLAIR hypointensities with some reports of cortical T2/FLAIR hyperintensities and restricted diffusion. Cortical gadolinium enhancement is rarely seen and may point to the other diagnoses^10,11,12^. The subcortical T2/FLAIR hypointense lesions may result from the intracellular dehydration of the glial tissue, mineral deposition, and free radical accumulation, whereas cortical hyperintense T2/FLAIR lesions may reflect transient vasogenic edema^11,12^. EEG abnormalities of aphasic status epilepticus includes focal discharges, lateralized periodic discharges (LPDs), and slow waves in the left frontal, temporal, and parietal cortices^2^. Nevertheless, some patients are only presented with slow background activity and intermittent diffuse theta and delta activities^3,13^.

The pathogenesis of hyperglycemia-induced seizure is not clearly defined. Several hypotheses have been proposed, including:

- Nonketotic hyperglycemia is a proconvulsant state that reduces the level of gamma-aminobutyric acid (GABA) and activates the ATP-sensitive potassium (K_ATP_) channel in the neocortex, while ketoacidosis in type 1 DM has an anti-seizure action due to intracellular acidosis increasing glutamic acid and decarboxylase activity, thus leading to increased level of GABA^4,7^;
- Decreased local blood flow from reduced levels of nitric oxide and hyperviscosity leading to focal ischemia, blood-brain barrier injury, and brain edema^3^;
- Decreased expression of Aquaporin-4 (AQP-4) predisposes to the development of vasogenic edema^11^;
- Small previous malformations of cortical development, such as cortical dysplasia, as well as old vascular or traumatic focal lesions, may be part of the predisposing factors for seizures^11,14^.

Although it has been acknowledged that hyperglycemia-induced seizures respond well to intensive insulin therapy and fluid replacement^3,4,13^, many researchers have found that some aphasic seizures remained uncontrolled until the addition of anti-seizure medications (ASMs)^2,7,8,9,14,15,16^. Therefore, long-term EEG monitoring may be needed in patients suspected with this condition who are unresponsive to intensive glycemic control. ASMs indicated for focal seizures, such as phenytoin, carbamazepine, and levetiracetam, could be used with reports of good outcomes such as our patient’s. Finally, the mainstay of treatment is long-term control of hyperglycemia to prevent recurrent seizures and other systemic complications of diabetes mellitus.
